# Long‐Term Efficacy and Safety of Mavacamten in Chinese Patients With Obstructive Hypertrophic Cardiomyopathy: Week 78 Results From the EXPLORER‐CN Study

**DOI:** 10.1161/JAHA.125.046251

**Published:** 2026-05-25

**Authors:** Zhuang Tian, Xiaoyan Li, Liwen Li, Qing Zhang, Jian’an Wang, Yunqi Shi, Daoquan Peng, Wei Ma, Ping Yang, Xiang Cheng, Wei Jin, Fang Wang, Yimeng Xie, Beth Pan, Victoria Florea, Shuyang Zhang

**Affiliations:** ^1^ Peking Union Medical College Hospital Chinese Academy of Medical Sciences and Peking Union Medical College Beijing China; ^2^ Renmin Hospital of Wuhan University, Hubei General Hospital Wuhan China; ^3^ Guangdong Cardiovascular Institute, Guangdong Provincial People’s Hospital, Guangdong Academy of Medical Sciences Southern Medical University Guangzhou China; ^4^ West China Hospital, Sichuan University Chengdu China; ^5^ The Second Affiliated Hospital of Zhejiang University School of Medicine Hangzhou China; ^6^ The People’s Hospital of Liaoning Province Shenyang China; ^7^ The Second Xiangya Hospital of Central South University Changsha China; ^8^ Peking University First Hospital Beijing China; ^9^ China‐Japan Union Hospital of Jilin University Changchun China; ^10^ Union Hospital, Tongji Medical College Huazhong University of Science and Technology Wuhan China; ^11^ Ruijin Hospital, Shanghai Jiaotong University School of Medicine Shanghai China; ^12^ Beijing Hospital, National Center of Gerontology and Institute of Geriatric Medicine, Chinese Academy of Medical Sciences Beijing China; ^13^ Bristol Myers Squibb Shanghai China; ^14^ Bristol Myers Squibb Beijing China; ^15^ Bristol Myers Squibb Princeton NJ USA

**Keywords:** long‐term outcomes, mavacamten, obstructive hypertrophic cardiomyopathy, Heart Failure

## Abstract

**Background:**

Long‐term efficacy and safety of mavacamten in Chinese patients with obstructive hypertrophic cardiomyopathy are unknown.

**Methods:**

Patients who completed the 30‐week, double‐blind, placebo‐controlled treatment period in EXPLORER‐CN (A Study to Evaluate the Efficacy and Safety of Mavacamten in Chinese Adults With Symptomatic Obstructive HCM), with no active safety concerns, were eligible to enter the long‐term extension period to receive 48‐week mavacamten treatment. Patients previously on mavacamten continued mavacamten (dose at week 30; mavacamten–mavacamten group); patients previously on placebo received mavacamten (starting dose, 2.5 mg once daily; placebo–mavacamten group). Key efficacy end points included change from baseline in echocardiographic measures, New York Heart Association functional class, 23‐item Kansas City Cardiomyopathy Questionnaire Clinical Summary Score, and cardiac biomarkers through week 78. Analyses were descriptive; no between‐group hypothesis testing was performed.

**Results:**

Seventy‐nine patients (mean age, 51.6 years; 27.8% women) entered the long‐term extension period (mavacamten–mavacamten, n=54; placebo–mavacamten, n=25). In the mavacamten–mavacamten group, numerical improvements in Valsalva and resting left ventricular outflow tract peak gradients were maintained through week 78 (mean change from baseline, −73.0 mm Hg [95% CI, −86.4 to −59.5]; and −56.3 mm Hg [95% CI, −67.1 to −45.5], respectively). Numerical improvements were also observed for other echocardiographic parameters, New York Heart Association class, Kansas City Cardiomyopathy Questionnaire Clinical Summary Score, and cardiac biomarkers through week 78. In the placebo–mavacamten group, numerical improvements with mavacamten in these parameters were noted from week 30 to week 78. Long‐term mavacamten treatment appeared well tolerated; left ventricular ejection fraction <50% was rare.

**Conclusions:**

In this exploratory long‐term extension study, 78‐week mavacamten treatment appeared well‐tolerated and was associated with numerical improvements from baseline in echocardiographic parameters, New York Heart Association class, patient‐reported health status, and cardiac biomarkers in Chinese patients with obstructive hypertrophic cardiomyopathy.

**Registration:**

URL: https://www.clinicaltrials.gov; Unique Identifier: NCT05174416.

**Nonstandard Abbreviations and Acronyms**
CFB: change from baseline
CV: coefficient of variation
CYP: cytochrome P450
DBPC: double‐blind, placebo‐controlled
EXPLORER‐CN: A Study to Evaluate the Efficacy and Safety of Mavacamten in Chinese Adults With Symptomatic Obstructive HCM
EXPLORER‐HCM: Clinical Study to Evaluate Mavacamten (MYK‐461) in Adults With Symptomatic Obstructive Hypertrophic Cardiomyopathy
GMR: geometric mean ratio
HCM: hypertrophic cardiomyopathy
KCCQ‐23 CSS: 23‐item Kansas City Cardiomyopathy Questionnaire Clinical Summary Score
LAVI: left atrial volume index
LTE: long‐term extension
MAVA‐LTE: A Long‐Term Safety Extension Study of Mavacamten in Adults Who Have Completed EXPLORER‐HCM
NYHA: New York Heart Association
PIONEER‐OLE: Extension Study of Mavacamten in Adults With Symptomatic Obstructive Hypertrophic Cardiomyopathy Previously Enrolled in PIONEER
TEAE: treatment‐emergent adverse event
VALOR‐HCM: A Study to Evaluate Mavacamten in Adults With Symptomatic Obstructive HCM Who Are Eligible for Septal Reduction Therapy

**Clinical Perspective**

**What Is New?**

- Long‐term mavacamten treatment was associated with numerical improvements from baseline in echocardiographic parameters of cardiac structure and function, New York Heart Association functional class, patient‐reported health status, and cardiac biomarkers that were maintained up to week 78 in Chinese patients with obstructive hypertrophic cardiomyopathy, along with a manageable safety profile.

**What Are the Clinical Implications?**

- Our findings suggest that the long‐term efficacy and safety of mavacamten treatment extend to Chinese patients with symptomatic obstructive hypertrophic cardiomyopathy.
- Our results appear consistent with the global studies and provide support for the long‐term use of mavacamten in the Chinese population.

Hypertrophic cardiomyopathy (HCM) is a primary myocardial disorder characterized by left ventricular (LV) hypertrophy, hypercontractility, and diastolic dysfunction.^1^, ^2^ LV outflow tract (LVOT) obstruction is a major hallmark of obstructive HCM, identified in ≈70% of patients in a Western cohort with HCM^2^, ^3^, ^4^; obstructive HCM is also common among patients with HCM in China, with a rising prevalence over the past several decades.^5^, ^6^ LVOT obstruction is one of the key prognostic factors for patients with HCM and is associated with increased risks of atrial fibrillation (AF), stroke, heart failure (HF), disease progression, and death.^7^, ^8^

Mavacamten is a first‐in‐class cardiac myosin inhibitor that has been approved across 5 continents for the treatment of symptomatic New York Heart Association (NYHA) functional class II to III obstructive HCM.^9^, ^10^, ^11^, ^12^, ^13^ Mavacamten addresses the underlying pathophysiology of obstructive HCM by reducing the formation of excess myosin–actin cross‐bridges, which contributes to myocardial hypercontractility. Current guidelines recommend cardiac myosin inhibitors (such as mavacamten) for treatment of obstructive HCM in adults with persistent symptoms despite treatment with β blockers, verapamil, or diltiazem.^3^, ^14^ In the pivotal global phase 3 EXPLORER‐HCM (Clinical Study to Evaluate Mavacamten [MYK‐461] in Adults With Symptomatic Obstructive Hypertrophic Cardiomyopathy) trial, mavacamten significantly reduced LVOT gradients and improved exercise capacity, NYHA functional class, and patient‐reported health status compared with placebo in patients with symptomatic obstructive HCM through 30 weeks.^15^, ^16^ Treatment benefits of mavacamten in improving LVOT gradients and health status also extended to diverse populations, including Chinese and Japanese adults with obstructive HCM in the phase 3 EXPLORER‐CN (A Study to Evaluate the Efficacy and Safety of Mavacamten in Chinese Adults With Symptomatic Obstructive HCM; NCT05174416) and HORIZON‐HCM (A Study of Mavacamten in Obstructive Hypertrophic Cardiomyopathy) trials, respectively.^17^, ^18^

While clinical benefits of mavacamten were sustained over 180 weeks of treatment in the global patient population with obstructive HCM (EXPLORER‐Long Term Extension [LTE] cohort) in the MAVA‐LTE (A Long‐Term Safety Extension Study of Mavacamten in Adults Who Have Completed EXPLORER‐HCM) trial,^19^ the long‐term efficacy and safety profiles of mavacamten are unknown in the Chinese population. Mavacamten is the first‐in‐class and only cardiac myosin inhibitor available in China, where there is a rising incidence of HCM overall^5^, ^20^; therefore, understanding the long‐term treatment effects of mavacamten in this population is of clinical interest.^21^ The EXPLORER‐CN study was designed to include a long‐term extension (LTE) period, during which eligible patients who completed the preceding 30‐week double‐blinded, placebo‐controlled (DBPC) treatment period without active safety concerns all received mavacamten treatment for 48 weeks, followed by 8 or 20 weeks of posttreatment follow‐up.^17^, ^22^ In this publication, we report the data on key echocardiographic measures, clinical symptoms, patient‐reported health status, and cardiac biomarkers, as well as the safety profile up to week 78 of the EXPLORER‐CN trial.

This publication represents the first long‐term analysis of mavacamten treatment conducted exclusively in the Chinese population, with a focus on the long‐term effects of mavacamten treatment on changes in LVOT gradients and the reproducibility of treatment benefit in patients who crossed over from the placebo group.

## Methods

Bristol Myers Squibb's policy on data sharing is available online at https://www.bms.com/researchers‐and‐partners/independent‐research/data‐sharing‐request‐process.html.

### Study Design, Dosing, and Patients

Details of the DBPC treatment period of the EXPLORER‐CN study have been published previously.^17^, ^22^ In brief, the DBPC treatment period lasted 30 weeks, which was followed by an LTE treatment period of 48 weeks and posttreatment follow‐up period of 8 weeks (or 20 weeks for poor cytochrome P450 [CYP] 2C19 metabolizers) (Figure S1). This article reports the results of the LTE period up to week 78 since randomization.

Full inclusion and exclusion criteria for the EXPLORER‐CN trial have been described previously.^22^ Key inclusion criteria included age ≥18 years, confirmed diagnosis of obstructive HCM, unexplained LV hypertrophy with nondilated ventricular chambers in the absence of other cardiac conditions or systemic disease and maximal LV wall thickness of ≥15 mm (or ≥13 mm if familial HCM), peak LVOT gradient ≥50 mm Hg at rest or with Valsalva, LV ejection fraction (LVEF) ≥55%, and NYHA functional class II to III.

During the DBPC period, patients were randomized 2:1 to once‐daily mavacamten or placebo at 12 sites in China. Patients who completed the 30‐week DBPC treatment period and had no active safety concerns in the judgment of the investigator were eligible to enter the LTE period. During the LTE period, patients received mavacamten in a double‐blind manner until all patients completed 30‐week DBPC treatment and the 30‐week treatment database was locked, after which all patients received mavacamten in an open‐label manner. The total duration of mavacamten exposure during the LTE period was up to 48 weeks for all patients. During the LTE period, patients previously on mavacamten during the DBPC period continued with the dose received at week 30 (mavacamten–mavacamten group); patients previously on placebo received mavacamten at a starting dose of 2.5 mg once daily, which was adjusted according to a dose titration scheme based on site‐read LVEF and Valsalva LVOT gradient (placebo–mavacamten group). Permissible doses were 1, 2.5, 5, 10, and 15 mg. The prespecified criterion for temporary discontinuation of mavacamten during the LTE period was a resting LVEF of <50%.

This trial was conducted in accordance with the principles of the Declaration of Helsinki and Guideline for Good Clinical Practice. The protocol was approved by institutional review boards/ethics committees at each site. All patients provided written informed consent before entering the study.

### End Points

The current study reports 78 weeks of drug exposure (mavacamten–mavacamten group) or 48 weeks (weeks 30–78) of drug exposure for patients originally on placebo who crossed over to mavacamten (placebo–mavacamten group).

Key efficacy end points included change from baseline in echocardiographic parameters, NYHA functional class, 23‐item Kansas City Cardiomyopathy Questionnaire Clinical Summary Score (KCCQ‐23 CSS), and cardiac biomarkers (NT‐proBNP [N‐terminal pro‐B‐type natriuretic peptide] and hs‐cTnI [high‐sensitivity cardiac troponin I]) through end of treatment at week 78. Key echocardiographic parameters included Valsalva and resting peak LVOT gradients, LV mass index, left atrial volume index (LAVI), and lateral and septal E/e' ratio, which were based on core‐read echocardiography.

Safety end points included incidence of treatment‐emergent adverse events (TEAEs), serious TEAEs, adverse events of special interest, LVEF <50%, major adverse cardiac events, HF events, AF/atrial flutter, implantable cardioverter‐defibrillator therapy and resuscitated cardiac arrest, and ventricular tachyarrhythmias. Adverse events of special interest were defined as events of symptomatic overdose, outcomes of pregnancy, and LVEF ≤30% assessed by echocardiogram.

### Statistical Analysis

For the efficacy end point, the last nonmissing measurement taken before the first dose of assigned treatment in the DBPC period was used as baseline. Efficacy analyses were performed for all eligible patients who entered LTE and received at least 1 dose of mavacamten in the LTE period.

Continuous variables were summarized using descriptive statistics and presented as mean±SD or median (interquartile range) by visit time points. Categorical variables were presented in numbers and percentages by visit time points.

For the safety end point, the last nonmissing measurement taken before the first dose of mavacamten (ie, week 0 for the mavacamten–mavacamten group and week 30 for the placebo–mavacamten group) was taken as baseline. Safety analyses were performed for all patients who had received at least 1 dose of mavacamten in the study (ie, the safety analysis included data collected during the DBPC and LTE periods for the mavacamten–mavacamten group but only data collected during the LTE period in the placebo–mavacamten group). Safety analyses were summarized using descriptive statistics.

No formal hypothesis testing or between‐group comparisons were performed, and no imputation was performed for missing data in the LTE period. All changes reported in the article represent numerical changes. SAS version 9.4 (SAS Institute, Cary, NC) was used for all analyses.

## Results

### Baseline Characteristics

Among 81 patients with symptomatic obstructive HCM randomized in EXPLORER‐CN, 79 patients (mean age, 51.6±11.9 years; 22 [27.8%] women) completed the 30‐week DBPC treatment and entered the LTE period (Figure S1). The LTE population included all patients (n=54) initially randomized to mavacamten (mavacamten–mavacamten) and patients (n=25) who crossed over from placebo to mavacamten (placebo–mavacamten) after 30 weeks. The 2 patients excluded from the placebo–mavacamten group had discontinued the study prematurely during the 30‐week DBPC period.^17^ Key characteristics at baseline (ie, before the first dose of mavacamten) are shown in Table 1. A majority of patients were of NYHA class II (n=62 [78.5%]) and taking β blockers as background HCM therapy (n=70 [88.6%]). In addition, a majority were intermediate metabolizers (n=39 [49.4%]) and normal metabolizers (n=32 [40.5%]) of CYP2C19; 8 (10.1%) patients were CYP2C19 poor metabolizers. Mean Valsalva LVOT gradient was 106.8±43.2 mm Hg, and resting LVOT gradient was 74.6±35.1 mm Hg before the first dose of mavacamten in the mavacamten–mavacamten group; the corresponding values before the first dose of mavacamten in the placebo–mavacamten group were 116.3 (52.2) mm Hg and 77.1 (39.6) mm Hg, respectively. Mean KCCQ‐23 CSSs were 82.4±16.9 and 81.3±16.3 in the mavacamten–mavacamten and the placebo–mavacamten groups, respectively.

**Table 1:** Baseline Demographics and Characteristics

Characteristics	Mavacamten–mavacamten (n=54)	Placebo–mavacamten (n=25)	All patients in LTE (N=79)
Age, y	52.4±12.1	50.1±11.7	51.6±11.9
Sex, female	13 (24.1)	9 (36.0)	22 (27.8)
Body mass index, kg/m^2^	25.2±3.5	25.5±3.4	25.3±3.4
NYHA functional class			
Class I	0	2 (8.0)	2 (2.5)
Class II	44 (81.5)	18 (72.0)	62 (78.5)
Class III	10 (18.5)	5 (20.0)	15 (19.0)
CYP2C19 metabolizer phenotype			
Normal metabolizer	23 (42.6)	9 (36.0)	32 (40.5)
Intermediate metabolizer	24 (44.4)	15 (60.0)	39 (49.4)
Poor metabolizer	7 (13.0)	1 (4.0)	8 (10.1)
Background HCM therapy*			
β blockers	48 (88.9)	22 (88.0)	70 (88.6)
Calcium channel blockers†	4 (7.4)	2 (8.0)	6 (7.6)
Other	2 (3.7)	1 (4.0)	3 (3.8)

Data are mean±SD or number (percentage). Baseline was defined as the last nonmissing measurement taken before the first dose date of mavacamten. CYP2C19 indicates cytochrome P450 2C19; HCM, hypertrophic cardiomyopathy; LTE, long‐term extension; and NYHA, New York Heart Association.

* Based on case report form.

^†^ Includes verapamil and diltiazem only.

All 79 patients who entered the LTE period completed the full course of study treatment and the study. The mavacamten dose received at specified visit time points is summarized in Table S1, and the final mavacamten dosing by CYP2C19 phenotype is shown in Table S2. By the last drug dispensing visit, a majority of patients were receiving mavacamten at the 5‐ or 10‐mg doses, regardless of CYP2C19 phenotype. At week 78, predose mavacamten concentration was ≤700 ng/mL in most patients (79.6%) in the mavacamten–mavacamten group; the only patient with predose mavacamten concentration ≥1000 ng/mL was a CYP2C19 poor metabolizer. In the placebo–mavacamten group, 72.0% of patients had predose mavacamten concentration ≤700 ng/mL, and 1 patient with predose mavacamten concentration ≥1000 ng/mL was a CYP2C19 intermediate metabolizer.

### Long‐Term Efficacy

**Figure 1:**
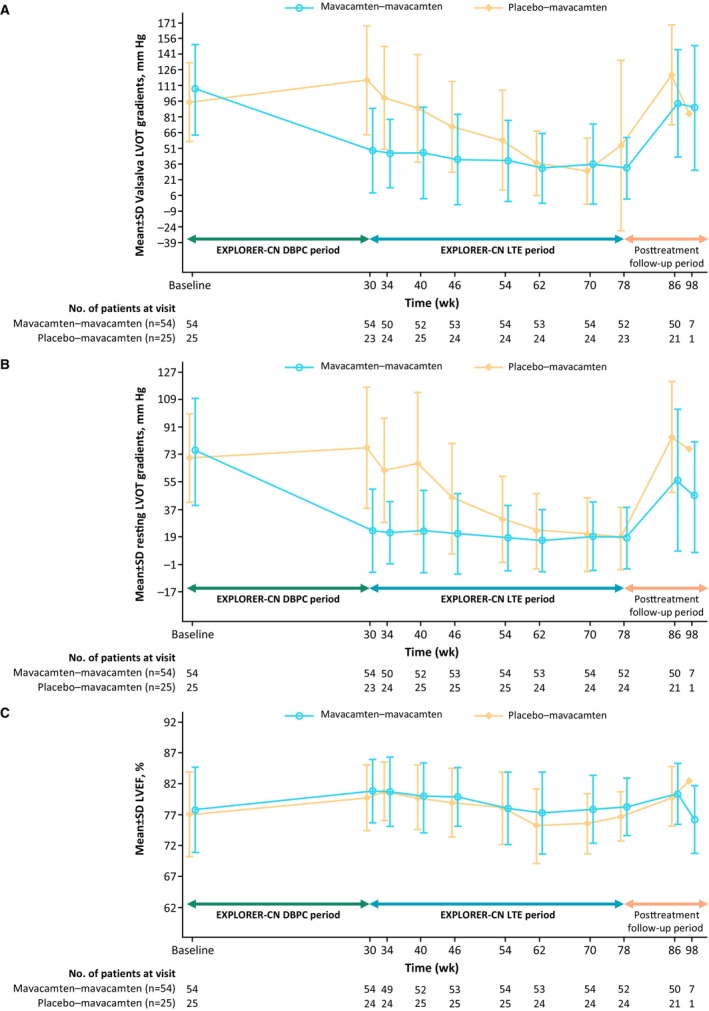
Echocardiographic parameters over time of (A) Valsalva LVOT gradient, (B) resting LVOT gradient, and (C) LVEF. Data are shown as mean±SD. Baseline values represent those taken before the first dose of the double‐blinded study drug at the beginning of EXPLORER‐CN. DBPC indicates double‐blind, placebo‐controlled; EXPLORER‐CN, A Study to Evaluate the Efficacy and Safety of Mavacamten in Chinese Adults With Symptomatic Obstructive HCM; LTE, long‐term extension; LVEF, left ventricular ejection fraction; and LVOT, left ventricular outflow tract.

The improvements in Valsalva LVOT peak gradients observed during the 30‐week DBPC period were maintained in numeric terms through week 78 in the LTE period for the mavacamten–mavacamten group, with a mean change from baseline (CFB) of −57.9 (95% CI, −70.4 to −45.5) mm Hg at week 30 and −73.0 (95% CI, −86.4 to −59.5) mm Hg at week 78 (Figure 1A and Table 2). For the placebo–mavacamten group, numerical decreases in Valsalva LVOT gradients were noted in parallel with initiation of mavacamten from week 30 onward (mean change from weeks 30–78, −60.6 mm Hg [95% CI, −107.3 to −13.8]) (Table 2). Similar trends in reductions were also observed for resting LVOT gradients, with numerical decrease maintained from baseline through week 78 in the mavacamten–mavacamten group (mean CFB, −56.3 mm Hg [95% CI, −67.1 to −45.5]) and a decrease that started with mavacamten exposure from week 30 onward in the placebo–mavacamten group (mean change from weeks 30–78, −56.2 mm Hg [95% CI, −75.3 to −37.1]) (Figure 1B and Table 2).

**Table 2:** Change in Echocardiographic, Functional, and Laboratory Parameters

Parameters	Mavacamten–mavacamten			Placebo–mavacamten		
	DBPC period, baseline to week 30 (n=54)	LTE period, weeks 30–78 (n=52)	DBPC+LTE period, baseline to week 78 (n=52)	DBPC period, baseline to week 30 (n=25)	LTE period, weeks 30–78 (n=23)	DBPC+LTE period, baseline to week 78 (n=24)
Valsalva LVOT gradient, mm Hg	−57.9 (−70.4 to −45.5)	−16.7 (−26.5 to −6.8)	−73.0 (−86.4 to −59.5)	20.7 (0.6 to 40.7)	−60.6 (−107.3 to −13.8)	−39.4 (−76.4 to −2.4)
Resting LVOT gradient, mm Hg	−51.5 (−61.3 to −41.6)	−5.5 (−11.8 to 0.7)	−56.3 (−67.1 to −45.5)	6.4 (−8.5 to 21.2)	−56.2 (−75.3 to −37.1)	−50.2 (−62.3 to −38.1)
≥1 class of NYHA improvement, n (%)*	32 (59.3)	36 (66.7)	36 (66.7)	4 (16.0)	15 (60.0)	15 (60.0)
NT‐proBNP, ng/L, GMR (CV%)	0.2 (95.7)	0.8 (88.4)	0.1 (125.3)	0.9 (55.1)	0.1 (172.2)	0.1 (131.9)
hs‐cTnI, ng/L, GMR (CV%)	0.4 (47.3)	0.8 (45.8)	0.4 (53.4)	1.2 (52.8)	0.4 (66.0)	0.5 (57.9)
KCCQ‐23 CSS	5.7 (1.5 to 9.9)	1.1 (−0.9 to 3.1)	7.1 (2.8 to 11.4)	−5.4 (−10.4 to −0.4)	7.3 (1.7 to 12.9)	1.9 (−5.0 to 8.7)
Change in additional echocardiographic parameters						
LVEF, %	3.0 (1.0 to 5.1)	−2.5 (−3.7 to −1.3)	0.1 (−1.6 to 1.8)	2.8 (0.6 to 5.0)	−2.9 (−5.2 to −0.7)	−0.2 (−2.7 to 2.4)
Septal E/e' ratio	−5.9 (−7.4 to −4.4)	0.3 (−1.0 to 1.6)	−5.6 (−7.3 to −3.8)	−0.6 (−2.7 to 1.5)	−5.1 (−7.9 to −2.4)	−5.8 (−7.9 to −3.8)
Lateral E/e' ratio	−4.2 (−5.7 to −2.7)	0.6 (−0.3 to 1.5)	−3.7 (−5.0 to −2.4)	−0.1 (−2.4 to 2.1)	−5.9 (−9.2 to −2.5)	−6.2 (−8.6 to −3.7)
LV mass index, g/m^2^	−25.4 (−33.8 to −17.0)	−6.2 (−13.7 to 1.3)	−29.8 (−38.3 to −21.3)	15.5 (−3.9 to 34.9)	−44.2 (−69.1 to −19.2)	−27.2 (−43.4 to −11.0)
LAVI, mL/m^2^	−10.8 (−13.5 to −8.2)	−1.3 (−3.2 to 0.7)	−12.3 (−15.0 to −9.7)	−3.7 (−6.9 to −0.5)	−11.9 (−16.5 to −7.4)	−16.1 (−21.4 to −10.8)
LVESVI, mL/m^2^	−2.5 (−4.1 to −1.0)	1.1 (0.2 to 2.1)	−1.0 (−2.2 to 0.1)	−2.9 (−5.1 to −0.7)	0.8 (−1.3 to 2.8)	−2.0 (−4.7 to 0.6)
LVEDVI, mL/m^2^	−4.3 (−7.6 to −1.0)	−0.5 (−3.7 to 2.6)	−4.7 (−7.8 to −1.7)	−5.5 (−11.4 to 0.4)	−2.9 (−8.3 to 2.5)	−8.3 (−15.8 to −0.8)
LVSVI, mL/m^2^	−1.6 (−4.2 to 1.0)	−1.5 (−4.1 to 1.0)	−3.4 (−6.0 to −0.8)	−2.8 (−7.4 to 1.7)	−3.3 (−7.4 to 0.7)	−6.2 (−11.7 to −0.7)
LVED interventricular septal thickness, mm	−2.4 (−3.2 to −1.7)	−1.1 (−1.9 to −0.3)	−3.5 (−4.2 to −2.8)	0.5 (−1.5 to 2.5)	−4.0 (−6.2 to −1.7)	−3.4 (−5.2 to −1.7)
LVED posterior wall thickness, mm	−0.5 (−1.0 to 0.0)	0.5 (0.0 to 1.0)	0.0 (−0.6 to 0.6)	1.2 (0.2 to 2.3)	−1.3 (−2.9 to 0.3)	−0.1 (−1.2 to 1.0)
LVED maximal wall thickness, mm	−2.5 (−3.3 to −1.7)	−0.7 (−1.5 to 0.0)	−3.2 (−4.1 to −2.4)	0.9 (−1.0 to 2.7)	−3.7 (−5.9 to −1.6)	−2.8 (−4.6 to −0.9)

Data are mean (95% CI), unless otherwise specified. The 95% CIs are based on normal approximation, unless otherwise specified. Efficacy baseline is defined as the last nonmissing measurement taken before the first dose date of double‐blinded study drug. CV indicates coefficient of variation; DBPC, double‐blind, placebo‐controlled; GMR, geometric mean ratio; hs‐cTnI, high‐sensitivity cardiac troponin I; KCCQ‐23 CSS, 23‐item Kansas City Cardiomyopathy Questionnaire Clinical Summary Score; LAVI, left atrial volume index; LTE, long‐term extension; LV, left ventricular; LVED, LV end‐diastolic; LVEDVI, LV end‐diastolic volume index; LVEF, LV ejection fraction; LVESVI, LV end‐systolic volume index; LVOT, LV outflow tract; LVSVI, LV stroke volume index; NT‐proBNP, N‐terminal pro‐B‐type natriuretic peptide; and NYHA, New York Heart Association.

* Percentages for post–week 30 visits are based on the denominator of n=54 in the mavacamten–mavacamten group and n=25 in the placebo–mavacamten group at week 30, which included only patients with nonmissing data at week 30. The Clopper–Pearson method was used for the 95% CIs.

In concordance with the improvements in LVOT obstruction noted above, long‐term numerical improvements in diastolic function were also noted, as assessed by echocardiography. Both septal and lateral E/e' ratio decreased numerically from baseline to week 30 in the mavacamten–mavacamten group (mean CFB, −5.9 [95% CI, −7.4 to −4.4] and −4.2 [95% CI, −5.7 to −2.7] for septal and lateral E/e', respectively) and were maintained through week 78 (mean CFB, −5.6 [95% CI, −7.3 to −3.8] and −3.7 [95% CI, −5.0 to −2.4], respectively), whereas numerical decreases in these measures were seen from week 30 to week 78 in the placebo–mavacamten group (mean change from weeks 30–78, −5.1 [95% CI, −7.9 to −2.4] and −5.9 [95% CI, −9.2 to −2.5], respectively) (Table 2). Numerical reductions from baseline to week 30 (mean CFB, −10.8 mL/m^2^ [95% CI, −13.5 to −8.2]) in LAVI were maintained through week 78 (mean CFB, −12.3 mL/m^2^ [95% CI, −15.0 to −9.7]) in the mavacamten–mavacamten group, whereas in the placebo–mavacamten group, numerical reductions were seen from week 30 to 78 (mean change from weeks 30–78, −11.9 mL/m^2^ [95% CI, −16.5 to −7.4]). Changes in other echocardiographic measures of cardiac structure and diastolic function are shown in Table 2. There were no obvious changes in mean LVEF to week 78 both in the mavacamten–mavacamten group (CFB, 0.1% [95% CI, −1.6 to 1.8]) and in the placebo–mavacamten group (CFB, −0.2% [95% CI, −2.7 to 2.4]) (Figure 1C). Overall, mean LVEF remained >60% with mavacamten treatment across all time points in both groups.

NYHA class continued to improve over time with mavacamten treatment. In the mavacamten–mavacamten group, 32 of 54 (59.3%) patients had improved by at least 1 class from baseline at week 30, which further increased to 36 of 54 (66.7%) patients at week 78 (Table 2). In the placebo–mavacamten group, 15 of 25 (60.0%) patients had improved by at least 1 class from baseline at week 78, while only 4 of 25 (16.0%) patients did so at week 30. As a result, approximately half of the patients were NYHA class I at week 78 (28 patients [51.9%]) in the mavacamten–mavacamten group and 10 patients [40.0%] in the placebo–mavacamten group.

Mavacamten treatment was also associated with continued improvements in patient‐reported health status: numerical increases in KCCQ‐23 CSS at week 30 (mean CFB, 5.7 [95% CI, 1.5–9.9]) were maintained through week 78 (mean CFB, 7.1 [95% CI, 2.8–11.4]) in the mavacamten–mavacamten group. In the placebo–mavacamten group, KCCQ‐23 CSS decreased numerically from baseline to week 30 (mean CFB, −5.4 [95% CI, −10.4 to −0.4]) but increased numerically from week 30 onward through week 78 (mean change from weeks 30–78, 7.3 [95% CI, 1.7 to 12.9]) (Table 2).

**Figure 2:**
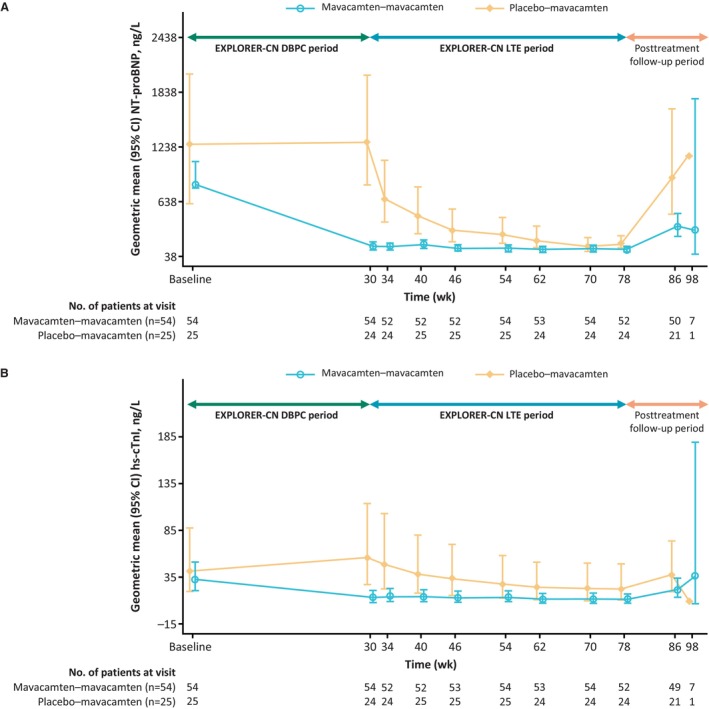
Cardiac biomarker levels over time of (A) NT‐proBNP and (B) hs‐cTnI. Data are shown as geometric mean (95% CI). Baseline values represent those taken before the first dose of the double‐blinded study drug at the beginning of EXPLORER‐CN. DBPC indicates double‐blind, placebo‐controlled; EXPLORER‐CN, A Study to Evaluate the Efficacy and Safety of Mavacamten in Chinese Adults With Symptomatic Obstructive HCM; hs‐cTnI, high‐sensitivity cardiac troponin I; IQR, interquartile range; LTE, long‐term extension; and NT‐proBNP, N‐terminal pro‐B‐type natriuretic peptide.

Furthermore, the numerical reduction from baseline to week 30 observed for NT‐proBNP levels (geometric mean ratio [GMR], 0.2; coefficient of variation [CV] %, 95.7) was maintained through week 78 (GMR, 0.1; CV%, 125.3) in the mavacamten–mavacamten group (Figure 2A; Table 2; Table S3). Conversely, no changes were seen in the placebo–mavacamten group from baseline to week 30; numerical decreases in NT‐proBNP levels were observed only from week 30 onward following mavacamten exposure (GMR, 0.1; CV%, 172.2). At week 78, the geometric mean (CV%) values were 108.7±103.2 and 176.9±121.3 in the mavacamten–mavacamten and the placebo–mavacamten groups, respectively. Similar trends were noted for changes in hs‐cTnI levels from baseline to week 30 (GMR, 0.4; CV%, 47.3) in the mavacamten–mavacamten group, with numerical decreases seen as early as week 4,^17^ which was maintained throughout the LTE period to week 78 (GMR, 0.4; CV%, 53.4). In the placebo–mavacamten group, there were no changes in hs‐cTnI levels from baseline to week 30, and hs‐cTnI levels started decreasing at week 30 after exposure to mavacamten (GMR, 0.4; CV%, 66.0) (Figure 2B; Table 2; Table S3). At week 78, the geometric mean (CV%) values of hs‐cTnI were 11.7±279.6 and 23.1±514.8 in the mavacamten–mavacamten and the placebo–mavacamten groups, respectively.

Analysis of efficacy measures by CYP2C19 metabolizer phenotypes showed largely consistent outcomes across normal, intermediate, and poor metabolizers, although interpretation is limited by small subgroup size (data not shown).

### Long‐Term Safety

Safety was assessed throughout the period of exposure to mavacamten, that is, during the DBPC and LTE period for the mavacamten–mavacamten group and during the LTE period for the placebo–mavacamten group; the outcomes are summarized in Table 3. During exposure to mavacamten, 345 TEAEs were reported in 53 patients (98.1%) in the mavacamten–mavacamten group, and 104 TEAEs were reported in 19 patients (76.0%) in the placebo–mavacamten group. Most TEAEs were of mild or moderate severity. Overall, the rates of TEAEs (related or not related to mavacamten) were comparable across CYP2C19 metabolizer phenotypes, although interpretation is limited by small sample size (Table S4). The most common cardiovascular TEAE was intraventricular conduction defect, all of which were reported in the mavacamten–mavacamten group (6 patients [11.1%]), and ventricular extrasystoles, which were reported in 4 patients (7.4%) and 2 patients (8.0%) in the mavacamten–mavacamten and placebo‐mavacamten groups, respectively (Table S5). No cardiac failure events were reported. Other cardiovascular TEAEs are presented in Table S5.

**Table 3:** Summary of Safety* Results

Parameters	Mavacamten–mavacamten (n=54)	Placebo–mavacamten (n=25)
TEAEs†	345	104
Patients with any TEAEs†	53 (98.1)	19 (76.0)
Patients with serious TEAEs	7 (13.0)	1 (4.0)
Patients with drug‐related TEAEs‡	15 (27.8)	6 (24.0)
Patients with drug‐related serious TEAEs‡	0	0
Patients with treatment interruption due to TEAEs	2 (3.7)	1 (4.0)
Patients with treatment discontinuation due to TEAEs	0	0

Data are n or number (percentage). DBPC indicates double‐blind, placebo‐controlled; LTE, long‐term extension; and TEAE, treatment‐emergent adverse event.

* Safety was assessed during the DBPC and LTE period for the mavacamten–mavacamten group and during the LTE period for the placebo–mavacamten group.

^†^ TEAEs in the DBPC and LTE period were defined as adverse events that started or worsened after the first dose of mavacamten to the end of the study.

^‡^ As assessed by the investigator and collected in the case report form; adverse events with missing relatedness were classified as related.

Overall, serious TEAEs were reported in 7 patients (13.0%) in the mavacamten–mavacamten group (2 CYP2C19 normal metabolizers and 5 intermediate metabolizers) and 1 patient (4.0%) in the placebo–mavacamten group (who was an intermediate CYP2C19 metabolizer) (Table 3; Table S4); none were judged by the investigator to be related to mavacamten. Among these, serious cardiovascular TEAEs were reported in 4 patients (5.1%) (Table S6). AF was the most common serious cardiac TEAE, reported in 2 patients (2.5%) overall, both of which were reported in the mavacamten–mavacamten group during the DBPC period; 1 of these patients had a history of AF. Other serious TEAEs are listed in Table S6.

A total of 3 patients (3.8%; 1 normal metabolizer and 2 intermediate metabolizers) reported 3 TEAEs that led to treatment interruption, all of which occurred during the LTE period. Of these, 1 was due to COVID‐19, 1 was due to musculoskeletal injury (both from the mavacamten–mavacamten group), and 1 was due to LV dysfunction in the placebo–mavacamten group; the latter 2 events were considered to be related to mavacamten. All patients eventually resumed the study drug, and there were no TEAEs leading to discontinuation of study treatment or deaths.

During the LTE period, 1 patient (1.9%) in the mavacamten–mavacamten group and 3 patients (12.0%) in the placebo–mavacamten group had LVEF <50%, as assessed by site‐read echocardiography. All 4 patients discontinued mavacamten per protocol‐defined interruption criteria. After 4 weeks of mavacamten discontinuation, LVEF for all 4 patients reverted back to ≥50% and mavacamten was resumed using the next lower dose, with no LVEF <50% reported thereafter.

There was no incidence of LVEF ≤30%. There were also no reports of major adverse cardiac events (including cardiovascular death, nonfatal stroke, and nonfatal myocardial infarction), HF‐related events, implantable cardioverter‐defibrillator therapy, resuscitated cardiac arrest, and ventricular tachyarrhythmias. None had reported new‐onset AF or atrial flutter during the LTE period.

## Discussion

Long‐term treatment with mavacamten, a first‐in‐class cardiac myosin inhibitor, was associated with numerical improvements in echocardiographic parameters, NYHA functional class, KCCQ‐23 CSS, and cardiac biomarkers in Chinese patients with obstructive HCM from baseline through 78 weeks. Not only were these treatment benefits maintained in the patient subgroup who continued on mavacamten from the DBPC period (mavacamten–mavacamten group), but the treatment benefits were also observed in the patient subgroup who crossed over from placebo to mavacamten (placebo–mavacamten group) from week 30 onward following initiation of mavacamten. These echocardiographic changes in response to long‐term mavacamten treatment observed in the Chinese patients were consistent with the global populations in the PIONEER‐OLE (Extension Study of Mavacamten in Adults With Symptomatic Obstructive Hypertrophic Cardiomyopathy Previously Enrolled in PIONEER), MAVA‐LTE (EXPLORER cohort), and VALOR‐HCM (A Study to Evaluate Mavacamten in Adults With Symptomatic Obstructive HCM Who Are Eligible for Septal Reduction Therapy) studies, despite a relatively shorter duration of our study compared with these global studies.^19^, ^23^, ^24^ While patients started on 2.5 mg mavacamten, a stable dose was achieved using individualized dose titration based on echocardiography, with similar efficacy results as the global population. Importantly, mavacamten was well tolerated, maintained mean LVEF >60% during the study without remarkable reduction following long‐term exposure to mavacamten in the Chinese population, and had a safety profile consistent with the global studies.

Long‐term treatment with mavacamten resulted in continued numerical reductions in both Valsalva and resting LVOT gradients accompanied by improvements in various measures of diastolic function through week 78, which provides evidence that the echocardiographic effects previously seen at week 30 were durable over the long term.^25^ While current standard pharmacologic therapies for obstructive HCM (such as β blockers and calcium channel blockers) offer symptomatic relief, their effect on diastolic function remains controversial.^26^ Further, these therapies do not address the underlying pathophysiological mechanisms of HCM, unlike mavacamten. The numerical improvements in diastolic measures following mavacamten exposure align with its mechanism of action reported in preclinical findings, namely, that inhibition of actin–myosin cross‐bridge formation has been shown to reduce LV stiffness and lower filling pressures, resulting in improved diastolic function.^27^, ^28^, ^29^ In our present study, numerical improvements in echocardiographic parameters of diastolic function, including LAVI and E/e' observed at week 30 were maintained through week 78 in the mavacamten–mavacamten group, indicating the durable treatment effects of mavacamten on echocardiographic changes. For the placebo–mavacamten group, numerical improvements in these echocardiographic parameters were observed from week 30 onward following the initiation of mavacamten exposure, consistent with the early treatment effects seen following mavacamten initiation in the mavacamten–mavacamten group during the DBPC period.^17^ Sustained improvements in these echocardiographic measures have also been noted with long‐term mavacamten treatment in the global studies, PIONEER‐OLE, MAVA‐LTE (EXPLORER cohort), and VALOR‐HCM.^19^, ^23^, ^24^ As elevated E/e' ratios and LAVI were predictors of adverse long‐term outcomes in HCM, including HF, AF, stroke, and sudden cardiac death,^30^, ^31^, ^32^, ^33^ improvements in these echocardiographic measures could potentially reduce the risk of adverse cardiovascular events in patients with HCM. In concordance, no major adverse cardiac events, HF‐related events, implantable cardioverter‐defibrillator therapy, resuscitated cardiac arrest, and ventricular tachyarrhythmias were reported during our study.

For both patient groups, favorable changes in echocardiographic parameters were accompanied by numerical reductions in hs‐cTnI to normal levels of ≤16 or ≤ 34 ng/L (depending on sex)^34^ at week 78, indicating reduced myocardial injury. NT‐proBNP, another cardiac biomarker, was also numerically reduced in both groups, particularly in the mavacamten–mavacamten group, which was lowered to normal levels by week 78.^35^ NT‐proBNP is a known indicator of cardiac wall stress and has been shown to be predictive of adverse cardiovascular outcomes, including HF and heart transplant–related death, in patients with HCM.^36^, ^37^ Considering that elevated levels of these cardiac biomarkers were associated with impaired LV function and adverse cardiovascular events,^36^ the benefit of mavacamten in lowering these biomarkers is particularly relevant for patients with obstructive HCM. However, in the absence of data on hard clinical outcomes such as hospitalization for HF, stroke, or cardiovascular death, interpretation of these biomarker trends should be considered as exploratory in nature. Nonetheless, it is worth noting that similar reductions in these cardiac biomarkers in response to mavacamten treatment were also observed in the global studies.^19^, ^23^

In general, long‐term mavacamten treatment up to week 78 was well tolerated, with a safety profile consistent with the DBPC period of EXPLORER‐CN and other studies of mavacamten in obstructive HCM.^17^, ^19^, ^23^, ^38^ Most TEAEs were of mild or moderate severity. None of the TEAEs led to study discontinuation, and no drug‐related serious TEAEs or deaths were reported. The profile of adverse events in Chinese patients with obstructive HCM is generally consistent with the global MAVA‐LTE trial (albeit with a different study duration), with recurrent arrhythmic and LV systolic function events that warrant ongoing monitoring during follow‐up. In addition, the rates of TEAEs and serious TEAEs were comparable across CYP2C19 metabolizer phenotypes, indicating that mavacamten administered using the dosing scheme in this study shows an acceptable safety profile in Chinese patients regardless of CYP2C19 metabolizer phenotypes, although this finding should be considered exploratory due to the small number of patients in each subgroup. There was no incidence of LVEF <50% during the DBPC period and double‐blind LTE period; 4 patients experienced LVEF <50% during the open‐label LTE period, resulting in temporary treatment discontinuation per protocol. LVEF subsequently recovered after treatment interruption, and all patients resumed mavacamten at a lower dose. No LVEF ≤30% occurred during the study. Nonetheless, longer‐term monitoring of cardiovascular events is important, including longer follow‐up data and real‐world studies, to clarify the cardiovascular safety profile of long‐term mavacamten use. Forthcoming data from a real‐world study of mavacamten in the Chinese population will help provide further validation to these findings.

Routine assessments with echocardiography are recommended to monitor the safety and efficacy of mavacamten. The stable outcomes observed in the present study, particularly in the mavacamten–mavacamten group, who had a total of 78 weeks of mavacamten exposure, suggest that long‐term efficacy and safety are achievable with the currently available doses and monitoring schedule once a stable dose has been achieved after the initiation and titration phases.

Given the open‐label LTE design of our study, our findings should be considered as hypothesis generating and interpreted in the context of some limitations. While the change from baseline data indicated improvements in efficacy measures from the beginning of the study, the open‐label LTE design prevents comparison with a control arm, which inherently limits causal inference. Also, all changes reported in the manuscript represent numerical changes. However, the direction of changes from weeks 30–78 in the mavacamten–mavacamten group are generally consistent with the changes observed during the DBPC period from baseline to week 30. Other limitations included relatively short duration of study, in comparison with the global studies with data out to week 180.^19^, ^23^ Observed recurrent arrhythmic and LV systolic function events would require longer‐term assessment to provide further validation of the cardiovascular safety profile of long‐term mavacamten use. Additionally, it should be noted that interpretation of findings with regard to CYP2C19 metabolizer phenotypes is limited due to the small number of patients in each subgroup. This study was conducted exclusively in Chinese patients with obstructive HCM; therefore, the findings are not generalizable to other populations. The cardiac parameters included here were assessed by echocardiographic imaging, though some parameters may be better characterized by cardiac magnetic resonance imaging. Analysis of cardiac magnetic resonance parameters on long‐term cardiac remodeling following mavacamten treatment is currently ongoing and will be presented in a separate paper.

In conclusion, our exploratory LTE study indicated that long‐term mavacamten treatment up to week 78 was associated with continued numerical improvements from baseline in echocardiographic parameters (including reduced LVOT peak gradients, improved E/e' ratio, and decreased LAVI), NYHA functional class, patient‐reported health status, and cardiac biomarkers in Chinese patients with obstructive HCM, while mean LVEF was maintained within normal range. Long‐term treatment with mavacamten appeared to be well tolerated, and most TEAEs were of mild or moderate severity. The safety profile of long‐term mavacamten in Chinese patients with obstructive HCM is broadly in line with the global MAVA‐LTE trial, although our study is of a relatively shorter duration, with recurrent arrhythmic and LV systolic function events that warrant ongoing monitoring during follow‐up. The incidence of LVEF <50% was low.

## Sources of Funding

This study was supported by Shanghai LianBio Development Co., Ltd and Bristol Myers Squibb.

## Disclosures

Y.X., B.P., and V.F. are employees of Bristol Myers Squibb and own stock in Bristol Myers Squibb. All authors reported medical writing support for the article funded by Bristol Myers Squibb.

## Supporting information

Tables S1–S6
Figure S1
