# Supplementary material for: Long‐Term Efficacy and Safety of Mavacamten in Chinese Patients With Obstructive Hypertrophic Cardiomyopathy: Week 78 Results From the EXPLORER‐CN Study
Source: J Am Heart Assoc. 2026 May 25;15(11):e046251. doi: 10.1161/JAHA.125.046251 (PMC13315352; doi:10.1161/JAHA.125.046251)
Supplement: Supplementary file 1 — Tables S1–S6 Figure S1 [file JAH3-15-e046251-s001.pdf]

SUPPLEMENTAL MATERIAL

Table S1: Mavacamten dose at specified visit time points

Visit, n (%)	0 mg	1 mg	2.5 mg	5 mg	10 mg	15 mg
Mavacamten-mavacamten group (n=54)						
Week 30	0	0	3 (5.6)	33 (61.1)	16 (29.6)	2 (3.7)
Week 70*	0	0	2 (3.7)	23 (42.6)	27 (50.0)	2 (3.7)
Placebo-mavacamten group (n=25)						
Week 74*	0	1 (4.0)	1 (4.0)	8 (32.0)	12 (48.0)	3 (12.0)

Data are n (%). All patients initiated mavacamten at 2.5 mg. By the last drug dispensing visit, most patients were taking mavacamten at the 5 or 10 mg doses: for the mavacamten-mavacamten group, 27 (50%) patients were taking 10 mg mavacamten and 23 (42.6%) patients were taking the 5 mg dose at week 70; for the placebo-mavacamten group, 12 (48.0%) patients were taking 10 mg mavacamten and 8 (32.0%) patients were taking the 5 mg dose.
*Indicates the last drug dispensing visit for each group. Total treatment period is 78 weeks.

Table S2: Mavacamten dose at specified visit time points by CYP2C19 phenotype

CYP2C19 phenotype	Normal		Intermediate		Poor	
**Final dosing at week 30,* n (%)**	**Mavacamten-mavacamten group (n=23)**	**Placebo-mavacamten group (n=9)**	**Mavacamten-mavacamten group (n=24)**	**Placebo-mavacamten group (n=15)**	**Mavacamten-mavacamten group (n=7)**	**Placebo-mavacamten group (n=1)**
1 mg	1 (4.3)	NA	0	NA	0	NA
2.5 mg	11 (47.8)	NA	2 (8.3)	NA	0	NA
5 mg	10 (43.5)	NA	14 (58.3)	NA	7 (100.0)	NA
10 mg	1 (4.3)	NA	7 (29.2)	NA	0	NA
15 mg	1 (4.3)	NA	1 (4.2)	NA	0	NA
**Final dosing at week 74,* n (%)**	**Mavacamten-mavacamten group (n=23)**	**Placebo-mavacamten group (n=9)**	**Mavacamten-mavacamten group (n=24)**	**Placebo-mavacamten group (n=15)**	**Mavacamten-mavacamten group (n=7)**	**Placebo-mavacamten group (n=1)**
1 mg	0	1 (11.1)	0	0	0	0
2.5 mg	0	1 (11.1)	2 (8.3)	0	0	0
5 mg	11 (47.8)	1 (11.1)	9 (37.5)	6 (40.0)	3 (42.9)	1 (100.0)
10 mg	11 (47.8)	4 (44.4)	12 (50.0)	8 (53.3)	4 (57.1)	0
15 mg	1 (4.3)	2 (22.2)	1 (4.2)	1 (6.7)	0	0

*The final dosing is measured based on the latest available dose in the data.

Table S3: Cardiac biomarker levels over time

Parameters, n (%)	Mavacamten-mavacamten			Placebo-mavacamten		
	Baseline (n=54)	Week 30 (n=54)	Week 78 (n=52)	Baseline (n=25)	Week 30 (n=24)	Week 78 (n=24)
NT-proBNP, ng/L, median (IQR)	870.5 (399.0, 1718.0)	125.5 (67.0, 281.0)	92.0 (50.0, 201.5)	1435.0 (532.0, 2713.0)	1291.5 (572.5, 2309.0)	185.0 (84.5, 385.5)
hs-cTnI, ng/L, median (IQR)	22.15 (8.59, 108.00)	10.20 (3.70, 29.51)	8.290 (3.81, 23.07)	40.20 (7.10, 99.00)	48.72 (16.50, 138.72)	12.60 (7.66, 45.42)

hs-cTnI, high-sensitivity cardiac troponin I; IQR, interquartile range; NT-proBNP, N-terminal prohormone of brain natriuretic peptide.

Table S4: Summary of TEAEs and serious TEAEs by CYP2C19 metabolizer phenotype

Parameters, n (%)	Mavacamten-mavacamten			Placebo-mavacamten		
	Normal metabolizer (n=23)	Intermediate metabolizer (n=24)	Poor metabolizer (n=7)	Normal metabolizer (n=9)	Intermediate metabolizer (n=15)	Poor metabolizer (n=1)
Any TEAE*	22 (95.7%)	24 (100%)	7 (100%)	6 (66.7%)	12 (80.0%)	1 (100%)
Drug-related TEAE†	4 (17.4%)	9 (37.5%)	2 (28.6%)	2 (22.2%)	3 (20.0%)	1 (100%)
Serious TEAEs	2 (8.7%)	5 (20.8%)	0	0	1 (6.7%)	0
Drug-related serious TEAEs†	0	0	0	0	0	0
TEAE leading to treatment interruption	1 (4.3%)	1 (4.2%)	0	0	1 (6.7%)	0

Safety was assessed during the DBPC and LTE period for the mavacamten-mavacamten group and during LTE period only for the placebo-mavacamten group.
*TEAEs in DBPC and LTE period are defined as adverse events that started or worsened after the first dose of mavacamten to the end of study.
†As assessed by the investigator and collected in the case report form; adverse events with missing relatedness are classified as related.
CYP2C19, cytochrome P450 2C19; DBPC, double-blind, placebo-controlled; LTE, long-term extension; and TEAE, treatment-emergent adverse event.

Table S5: Cardiovascular and nervous system disorders; TEAE summary (⩾2% in any arm)

Parameters*	Mavacamten-mavacamten (n=54)	Placebo-mavacamten (n=25)	All patients exposed to mavacamten (N=79)
Cardiac disorders	22 (40.7)	10 (40.0)	32 (40.5)
Intraventricular conduction defect	6 (11.1)	0	6 (7.6)
Ventricular extrasystoles	4 (7.4)	2 (8.0)	6 (7.6)
Angina pectoris	4 (7.4)	1 (4.0)	5 (6.3)
Cardiac discomfort	4 (7.4)	1 (4.0)	5 (6.3)
Ventricular tachycardia	4 (7.4)	1 (4.0)	5 (6.3)
Mitral valve incompetence	3 (5.6)	1 (4.0)	4 (5.1)
Palpitations	3 (5.6)	1 (4.0)	4 (5.1)
Atrioventricular block first degree	2 (3.7)	1 (4.0)	3 (3.8)
Atrial fibrillation	2 (3.7)	0	2 (2.5)
Nodal rhythm	2 (3.7)	0	2 (2.5)
Supraventricular extrasystoles	2 (3.7)	0	2 (2.5)
Cardiac aneurysm	1 (1.9)	1 (4.0)	2 (2.5)
Bundle branch block left	0	2 (8.0)	2 (2.5)
Pericardial effusion	0	2 (8.0)	2 (2.5)
Acquired left ventricle outflow tract obstruction	0	1 (4.0)	1 (1.3)
Extrasystoles	0	1 (4.0)	1 (1.3)
Left atrial enlargement	0	1 (4.0)	1 (1.3)
Left ventricular dysfunction	0	1 (4.0)	1 (1.3)
Pulmonary valve incompetence	0	1 (4.0)	1 (1.3)
Nervous system disorders	15 (27.8)	6 (24.0)	21 (26.6)
Dizziness	10 (18.5)	3 (12.0)	13 (16.5)
Hypoesthesia	3 (5.6)	1 (4.0)	4 (5.1)
Headache	1 (1.9)	2 (8.0)	3 (3.8)
Vascular disorders	6 (11.1)	1 (4.0)	7 (8.9)
Hypertension	4 (7.4)	0	4 (5.1)
Aortic arteriosclerosis	0	1 (4.0)	1 (1.3)

Data are n (%). Safety was assessed during the DBPC and LTE period for the mavacamten-mavacamten group and during the LTE period for the placebo-mavacamten group.
*By system organ class and preferred terms.
DBPC, double-blind, placebo-controlled; LTE, long-term extension; and TEAE, treatment-emergent adverse event.

Table S6: Serious TEAEs

Parameters*	Mavacamten-mavacamten (n=54)	Placebo-mavacamten (n=25)	All patients exposed to mavacamten (N=79)
Cardiac disorders	4 (7.4)	0	4 (5.1)
Atrial fibrillation	2 (3.7)	0	2 (2.5)
Angina pectoris	1 (1.9)	0	1 (1.3)
Atrial flutter	1 (1.9)	0	1 (1.3)
Cardiac discomfort	1 (1.9)	0	1 (1.3)
Sinus arrest	1 (1.9)	0	1 (1.3)
Sinus node dysfunction	1 (1.9)	0	1 (1.3)
Eye disorders	2 (3.7)	0	2 (2.5)
Amaurosis	1 (1.9)	0	1 (1.3)
Amaurosis fugax	1 (1.9)	0	1 (1.3)
Gastrointestinal disorders	2 (3.7)	0	2 (2.5)
Hemorrhoids	1 (1.9)	0	1 (1.3)
Large intestine polyp	1 (1.9)	0	1 (1.3)
Infections and infestations	1 (1.9)	0	1 (1.3)
Wound infection	1 (1.9)	0	1 (1.3)
Injury, poisoning, and procedural complications	1 (1.9)	0	1 (1.3)
Ankle fracture	1 (1.9)	0	1 (1.3)
Neoplasms benign, malignant, and unspecified	1 (1.9)	0	1 (1.3)
Papillary thyroid cancer	1 (1.9)	0	1 (1.3)
Vascular disorders	1 (1.9)	0	1 (1.3)
Hypotension	1 (1.9)	0	1 (1.3)
Congenital, familial, and genetic disorders	0	1 (4.0)	1 (1.3)
Hypertrophic cardiomyopathy	0	1 (4.0)	1 (1.3)

Data are n (%). Safety was assessed during the DBPC and LTE period for the mavacamten-mavacamten group and during the LTE period for the placebo-mavacamten group.
*By system organ class and preferred terms.
DBPC, double-blind, placebo-controlled; LTE, long-term extension; and TEAE, treatment-emergent adverse event.

Figure S1: Schematic diagram of EXPLORER-CN study design

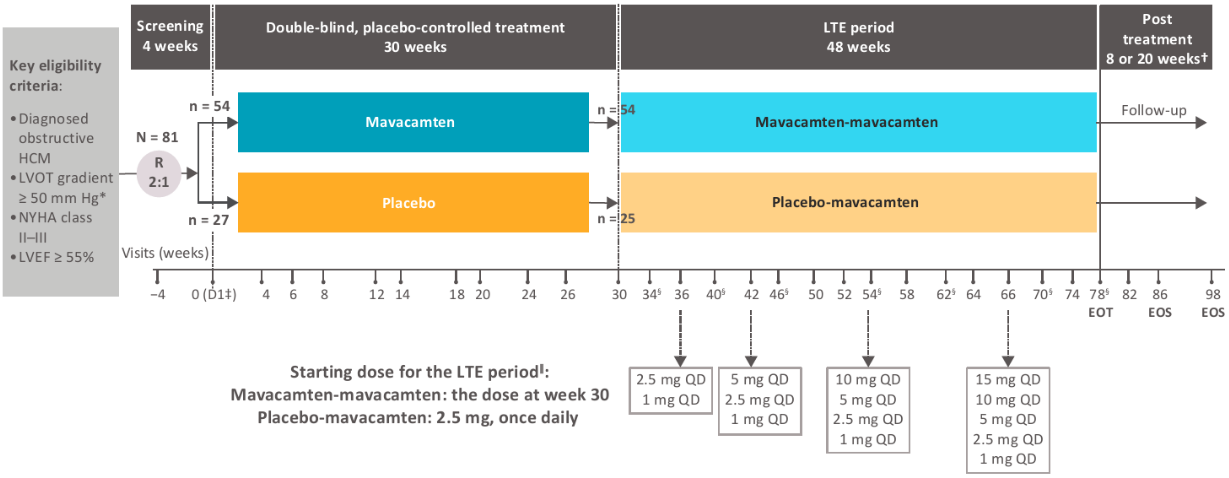

*LVOT peak gradient ≥50 mm Hg at rest or after Valsalva maneuver during screening. †Post-treatment follow-up period: 8 weeks (or 20 weeks for poor CYP2C19 metabolizer).
‡During the DBPC period, patients randomized to mavacamten received the drug at starting dose of 2.5 mg QD, with dose up-titration per protocol, if needed at week 8 to 14, and 20 based on central-read echocardiographic findings and plasma concentration; permissible doses: 1 mg to 2.5 mg to 5 mg to 10 mg, and 15 mg.
§Required visits for mavacamten-to-mavacamten patients during LTE period.
‖Dose adjustment during the LTE period was performed only for patients who were previously on placebo during the DBPC period, at weeks 36, 42, 54, and 66, based on site-read TTE on the day of visit. Patients who were previously on placebo started 2.5 mg mavacamten at the end of week 30. Patients previously on mavacamten continued on the dose received at week 30.
CYP2C19, cytochrome P450 2C19; DBPC, double-blind, placebo-controlled; EOS, end of study; EOT, end of treatment; HCM, hypertrophic cardiomyopathy; LTE, long-term extension; LVEF, left ventricular ejection fraction; LVOT, left ventricular outflow tract; NYHA, New York Heart Association; QD, once daily; R, randomization; TTE, transthoracic echocardiography.
